# Cinnamic Acid Derivatives as Cardioprotective Agents against Oxidative and Structural Damage Induced by Doxorubicin

**DOI:** 10.3390/ijms22126217

**Published:** 2021-06-09

**Authors:** Paulina Koczurkiewicz-Adamczyk, Katarzyna Klaś, Agnieszka Gunia-Krzyżak, Kamil Piska, Kalina Andrysiak, Jacek Stępniewski, Sławomir Lasota, Katarzyna Wójcik-Pszczoła, Józef Dulak, Zbigniew Madeja, Elżbieta Pękala

**Affiliations:** 1 Department of Pharmaceutical Biochemistry, Faculty of Pharmacy, Jagiellonian University Medical College, 30-688 Kraków, Poland; katarzyna.anna.klas@doctoral.uj.edu.pl (K.K.); kamil.piska@uj.edu.pl (K.P.); katarzynaanna.wojcik@uj.edu.pl (K.W.-P.); elzbieta.pekala@uj.edu.pl (E.P.); 2 Department of Bioorganic Chemistry, Chair of Organic Chemistry, Faculty of Pharmacy, Jagiellonian University Medical College, 30-688 Kraków, Poland; agnieszka.gunia@uj.edu.pl; 3 Department of Medical Biotechnology, Faculty of Biochemistry, Biophysics and Biotechnology, Jagiellonian University, 30-387 Kraków, Poland; kalina.andrysiak@doctoral.uj.edu.pl (K.A.); jacek.stepniewski@uj.edu.pl (J.S.); jozef.dulak@uj.edu.pl (J.D.); 4 Department of Cell Biology, Faculty of Biochemistry, Biophysics and Biotechnology, Jagiellonian University, 30-387 Kraków, Poland; slawomir.lasota@uj.edu.pl (S.L.); z.madeja@uj.edu.pl (Z.M.)

**Keywords:** doxorubicin, cardiotoxicity, combined therapy, cinnamic acid derivatives, cardioprotection, hiPSC-CM

## Abstract

Doxorubicin (DOX) is a widely used anticancer drug. However, its clinical use is severely limited due to drug-induced cumulative cardiotoxicity, which leads to progressive cardiomyocyte dysfunction and heart failure. Enormous efforts have been made to identify potential strategies to alleviate DOX-induced cardiotoxicity; however, to date, no universal and highly effective therapy has been introduced. Here we reported that cinnamic acid (CA) derivatives exert a multitarget protective effect against DOX-induced cardiotoxicity. The experiments were performed on rat cardiomyocytes (H9c2) and human induced-pluripotent-stem-cell-derived cardiomyocytes (hiPSC-CMs) as a well-established model for cardiac toxicity assessment. CA derivatives protected cardiomyocytes by ameliorating DOX-induced oxidative stress and viability reduction. Our data indicated that they attenuated the chemotherapeutic’s toxicity by downregulating levels of caspase-3 and -7. Pre-incubation of cardiomyocytes with CA derivatives prevented DOX-induced motility inhibition in a wound-healing assay and limited cytoskeleton rearrangement. Detailed safety analyses—including hepatotoxicity, mutagenic potential, and interaction with the hERG channel—were performed for the most promising compounds. We concluded that CA derivatives show a multidirectional protective effect against DOX-induced cardiotoxicity. The results should encourage further research to elucidate the exact molecular mechanism of the compounds’ activity. The lead structure of the analyzed CA derivatives may serve as a starting point for the development of novel therapeutics to support patients undergoing DOX therapy.

## 1. Introduction

Doxorubicin (DOX) is an anthracycline antibiotic used to treat various types of cancer, including solid tumors, hematologic malignancies, and soft tissue sarcoma [1,2]. Despite its great efficacy, its clinical use is limited by multiple side effects. Cardiotoxicity is one of the most serious. Patients can develop both acute and chronic cardiotoxic effects due to DOX. As a result of major functional and structural myocardial interruptions, arrhythmias, systolic dysfunction, heart failure, severe cardiomyopathy, or even death can develop [3,4].

Although many molecular pathways have been associated with the pathogenesis of DOX-induced cardiotoxicity, no universal model for the development of this phenomenon has been proposed. The most important cellular processes involved in cardiomyocyte dysfunction include increased oxidative stress and the generation of reactive oxygen species (ROS). The disruption of Ca^2+^ homeostasis, along with mitochondrial dysfunction and the apoptosis process seems to play an important role [4,5,6,7]. All of these may damage sarcomere structures, leading to disturbances in the myocyte dynamic and irreversible heart injury. Moreover, DOX metabolism may be involved in the development of cardiotoxicity [8]. The reduction of the carbonyl group to alcohol converts DOX into doxorubicinol (DOXol), which accumulates in the cardiomyocytes and contributes to their damage. Mechanistically, DOXol interferes with the sodium–potassium pump, leading to loss of ionic balance in the heart muscle [9].

To date, the efficacy of various drugs to alleviate fully developed DOX-induced cardiomyopathy was evaluated. One of the promising agents is dexrazoxane. The drug is used in clinical practice for patients treated with DOX due to metastatic breast cancer. Furthermore, dexrazoxane should be used with caution in children and adolescents receiving DOX because of possible harmful effects that may outweigh the benefits [10]. Angiotensin-converting enzyme inhibitors (such as enalapril, zofenopril, and lisinopril), statins, dietary supplements such as vitamin A, C, and E, coenzyme Q, N-acetyl cysteine, omega-3 fatty acids, and flavonoids were also evaluated [11,12,13,14]. Despite promising results based on preclinical studies, only some of these compounds entered clinical trials, and even fewer showed a positive effect on cardiac function and structure [15]. This further emphasizes the importance of implementing an effective cardioprotective strategy for DOX-treated patients.

In this paper, we focused on evaluating cinnamic acid (CA) amide derivatives as potential substances with cardioprotective activity. CA and cinnamide derivatives, both synthetic and derived from natural sources, were consequently reported to exert a wide range of biological activities, including anticancer, hepatoprotective, neuroprotective, angiogenic, and antidiabetic effects [16,17,18]. Their protective effects in acute myocardial ischemia were also reported [19]. In addition, some compounds were discussed as potential cardioprotective agents against DOX-induced cardiac injury. For example, in vivo studies showed that curcumin was effective in attenuating DOX-induced inflammation, apoptosis, and oxidative DNA damage [20]. In the model of H9c2 rat cardiomyocyte cells, p-coumaric acid significantly reduced DOX-induced ROS production [21]. Previously, we showed that CA derivatives, which were designed and synthetized by our group, exhibited chemosensitizing activity in DOX-treated A549 lung cancer cells. They were found to interact with active sites of the CBR1 enzyme and decrease DOXol formation [22]. 

Considering the above, we decided to perform a screening of 16 active CA derivatives in cardiomyocytes injured by DOX. Three compounds (**5**, **10**, and **15**) that exerted the most favorable safety profiles were subjected to further analyses. Their cardioprotective properties were evaluated in H9c2 rat cardiomyocyte cell lines and the hiPSC-CM model. We examined the ability of CA derivatives to alleviate DOX-induced disruption of important cellular physiological processes (i.e., oxidative stress, apoptosis, cell motility, and cytoskeleton morphology). In addition, an in-depth analysis of the safety profiles was performed for the compounds. Their hepatotoxic and mutagenic activity, respectively, as well as their interaction with the hERG channel, were excluded. 

Collectively, our data indicates that CA derivatives may act as protective agents in a DOX-induced cardiotoxicity model. We propose them to be potential therapeutics for the prevention of cancer-therapy-related cardiac dysfunction and heart failure.

## 2. Results

The compounds used for the study were biologically active CA (cinnamic acid) derivatives previously synthesized by our group, with proven inhibitory potential for the CBR1 enzyme [22,23,24,25,26]. This paper presents the cardioprotective activity of the three most active derivatives: **5**, **10,** and **15**. These were selected from among 16 compounds on the basis of preliminary analysis of their cardioprotective effects. The chemical structures of compounds **5** ((R,S)-(E)-N-(1-hydroxy-3-methylbutan-2-yl)cinnamamide), **10** ((E)-3-(4-chlorophenyl)-N-(1-hydroxy-2-methylpropan-2-yl)acrylamide), and **15** ((E)-1-(4-hydroxypiperidin-1-yl)-3-phenylprop-2-en-1-one) are presented in Table 1.

### 2.1. CA Derivatives Protected against DOX-Induced Cytotoxicity and Oxidative Stress in H9c2 Cells and hiPSC-CMs

**Figure 1:**
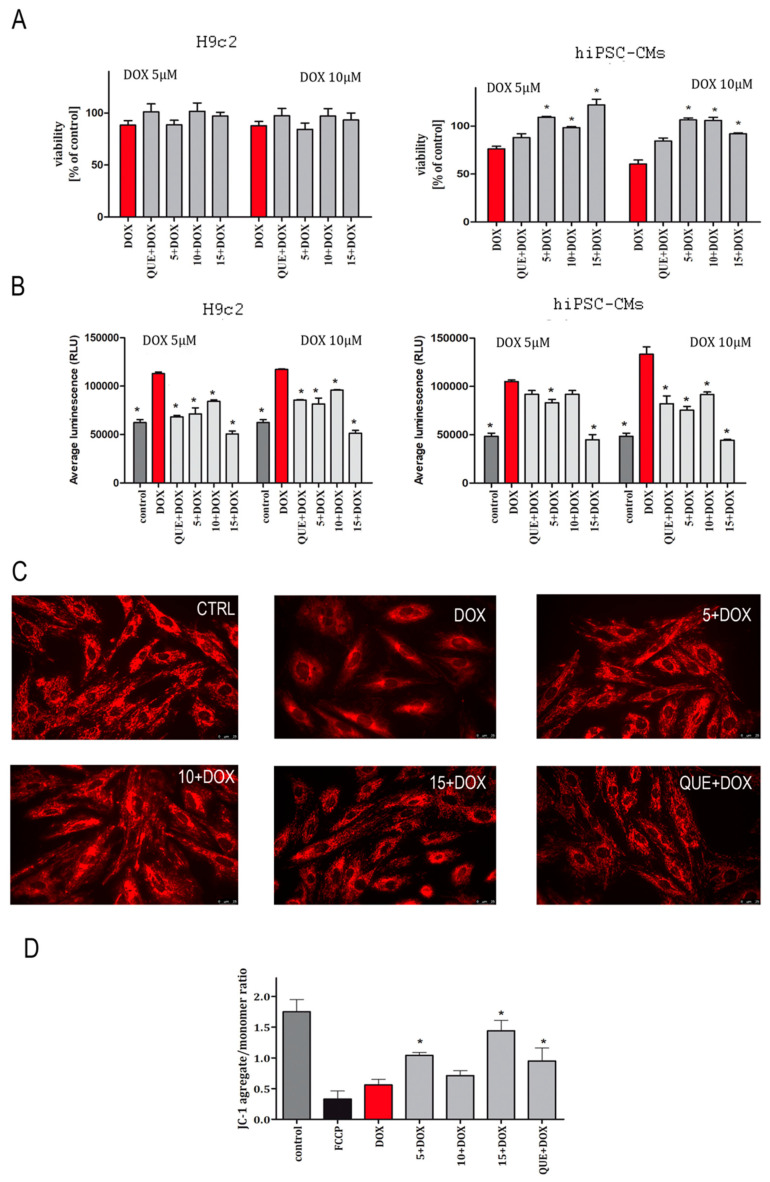
Protective effect of compounds **5**, **10**, and **15** against doxorubicin-induced cytotoxicity (**A**), oxidative stress (**B**), and mitochondrial damage (**C**,**D**). The study was performed in two cellular models: H9c2 and hiPSC-CMs. Cells were cultured with tested compounds—**5**, **10**, **15** (25 µM), and quercetin (20 µM)—as a reference standard for 5 h. Then, the medium was removed, cells were incubated with doxorubicin (DOX, 5 or 10 µM) for 90 min, and the MTT (**A**) or ROS-Glo™ H_2_O_2_ (**B**) assays were performed. Values represent the viability of cells in the percentage of control (untreated cells) ± SEM of six replicates of three independent experiments (**A**) or average RLU (relative light unit) ± SEM of six replicates of three independent experiments (**B**). The RLU value increases in direct proportion to the level of oxidative stress generated by the cells. Statistical significance (*) was calculated relative to DOX (*p* < 0.05). The effect of cinnamic acid derivatives and doxorubicin (CA–DOX) co-treatment on mitochondrial morphology was analyzed via fluorescent photomicrographs representing MitoTracker fluorescent staining of H9c2 cells (**C**). Bar graph representing JC-1 fluorescent mitochondrial depolarization of H9c2 cells’ membrane potential after CA–DOX treatment. Data are represented as two independent biological experiments, each with four technical replicates (*n* = 8). Results are expressed as the mean ± SEM (**D**).

In the conducted experiments, the cells were pre-incubated with the examined CA derivatives in concentrations of 25 µM for 5 h, then DOX was added to the culture medium at concentrations of 5 and 10 µM for the next 90 min. Quercetin (**QUE**), a flavonoid with well-described cardioprotective activity, was used as a reference [27,28,29]. MTT and ROS-Glo H_2_O_2_ assays were performed in order to measure the ability of CA to prevent DOX-induced damage in cardiomyocytes. Our research has shown that both used cellular models are characterized by different cellular responses to the tested compounds (Figure 1). In the case of the cells’ viability measurements, the protective effects of the tested compounds were not confirmed for the H9c2 cardiomyocyte cell line. In the case of hiPSC-CMs, at both tested concentrations of the damaging compound (DOX), a statistically significant protective effect was noted for the tested compounds (**5**, **10**, and **15**) and for the reference (**QUE**) (Figure 1A). ROS-Glo H_2_O_2_ assay showed that all CA derivatives possess protective activity against DOX-induced oxidative stress in both cellular models. The exceptions were compound **10** and **QUE**, for which no protective effect was observed against damage from a lower DOX concentration (Figure 1B). The influence of the compounds on the condition of mitochondria (morphology and potential of the mitochondrial membrane) was also examined. MitoTracker staining revealed mitochondrial morphological changes in DOX-treated cells. The tested CA derivatives counteracted the damage caused by DOX. Cells incubated in CA–DOX regimens had unchanged mitochondrial morphology. Compound **10** showed the lowest protective potential (Figure 1C). We next examined the effect of DOX and CA–DOX treatment on cell mitochondrial membrane potential, measured by JC-1 staining. DOX disrupted the mitochondrial membrane potential in the initial stages of the experiment, as indicated by the alterations in JC-1 dye aggregation (as with reference compound FCCP). DOX treatment for 3 h caused a significant reduction in aggregate/monomer JC-1 ratio, as indicated by the depolarized MMP. Co-treatment with CA **5**, **15** and **QUE** resulted in gradual recovery of MMP, as indicated by the increase in the aggregate/monomer ratio (Figure 1D). Only compound **10** did not cause statistically significant improvement in the state of the mitochondrial membrane potential.

### 2.2. CA Derivatives Protected against DOX-Induced Apoptosis in H9c2 Cells and hiPSC-CMs

**Figure 2:**
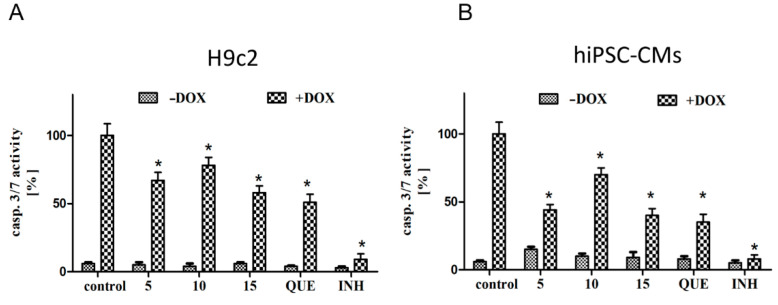
The protective effects of compounds **5**, **10**, and **15** against doxorubicin-induced apoptosis. (**A**) represents the results for H9c2 and (**B**) for hiPSC-CMs. Cells were seeded into 96-well plates; after 24 h, cells were pre-incubated in the presence of the compounds (**5**, **10**, **15**, and **QUE**) for 24 h, and then DOX was added for the next 24 h. Luminescent detection of caspase-3 and -7 was performed according to the manufacturer’s specifications. Caspase-3/7 activation results are expressed as a percentage (mean value for condition × 100/control) ± SEM, *n* = 9. Statistical significance (*) was calculated relative to control (*p* < 0.05) separately for DOX (–) and DOX (+) conditions. Cells were incubated with or without the wide-ranging caspase inhibitor (INH) Z-VAD-FMK at a concentration of 20 µM, in order to indicate the caspase-dependent mechanism of apoptosis.

The activity of caspase-3/7, major effector proteins of the apoptosis process, was measured. Cells were preincubated in the presence of CA derivatives (**5**, **10**, and **15**) for 24 h, and then DOX was added to the culture medium at a concentration of 0.5 µM in order to induce cell apoptosis. Treatment with DOX alone potentiated caspase-3/7 activity, while analyzed compounds did not exert pro-apoptotic activity in either experimental cellular model. Preincubation in the presence of CA derivatives at 25 µM markedly attenuated DOX-induced caspase-3/7 activity. The more profound cytoprotective effect on H9c2 cells was showed by compound **15** and **QUE,** which both reduced the apoptosis to 50% compared to cells treated with DOX (Figure 2A). A fundamental protective effect was observed for the hiPSC-CMs, particularly for compounds **5**, **15,** and **QUE,** which reduced caspase-3/7 levels to 35% compared to DOX-treated cells (Figure 2B). To investigate whether caspase played a role in the induction of apoptosis, the effect of pan-caspase inhibitor Z-VAD-FMK (20 µM) was used. For this purpose, cells were used in combination with or in the absence of the caspase inhibitor. The extent of apoptosis as determined by the percentage of activity of caspase-3/7 was significantly decreased in the presence of Z-VAD-FMK compared to the group receiving DOX alone (Figure 2A,B).

### 2.3. Preincubation in the Presence of CA Derivatives Protected H9c2 Cells against DOX-Induced Inhibition of Motility

**Figure 3:**
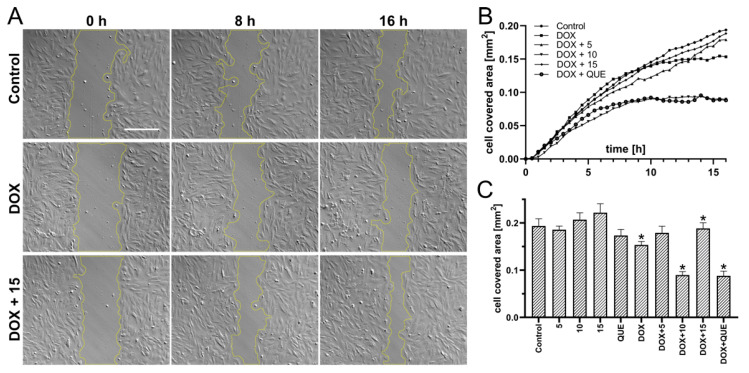
Effect of DOX in combination with compounds (**5**, **10**, and **15**) on the migration of rat cardiomyocytes in the wound-healing model. Cells were seeded at 1 × 10^5^ per well in 12-well plates and cultivated overnight in order to achieve 100% confluence, in the presence of the examined compound (25 µM). Artificial wounds were made manually with a pipette tip, after which the culture medium was replaced with a fresh one containing additional DOX (0.5 µM). Representative images show the most significant results of the wound-healing assay in vitro at indicated time steps. The area of the wound is outlined in order to be measured. Scale bar is 300 µm (**A**). Graph presenting the dynamics of wound closure. The average cell-covered area from multiple fields of view is presented with 30-min time steps. Only the most relevant conditions are presented, in order to increase the transparency of the results (**B**). Complete summary of analyzed conditions. Each bar shows the average cell-covered area within the entire experiment (after 16 h of recording) ± SEM; *n* > 10; * *p* < 0.05; statistical significance was determined using the Brown–Forsythe and Welch’s ANOVA tests, along with a post-hoc unpaired t-test with Welch’s correction (where **5**, **10**, **15**, **QUE,** and DOX were compared to control, and DOX + 5, DOX + 10, DOX + 15, and DOX + QUE were compared to DOX) (**C**).

DOX-induced decrease in the migration activity of cardiomyocytes can be avoided by pretreatment with CA derivatives. In this experiment, compound **15** had the best protective activity. Interestingly, neither compound **10** nor **QUE** showed any protective effect. Representative images showing the most crucial results of the wound-healing assay in vitro are presented in Figure 3A. Initial, intermediate, and final time steps are shown. The area of the wound was outlined in order to be measured. Wound closure in the presence of DOX (0.5 µM) progressed slower than in the control conditions. Treatment with compound **15** (25 µM) completely abolished this effect. The graph presents the dynamics of wound closure (Figure 3B,C). The averaged area from multiple fields of view is presented for subsequent 30-min time intervals. Only the most relevant conditions are presented, in order to increase the transparency of the results.

### 2.4. Compound **15** Protected Cardiomyocytes’ Cytoskeletons against DOX Damage

**Figure 4:**
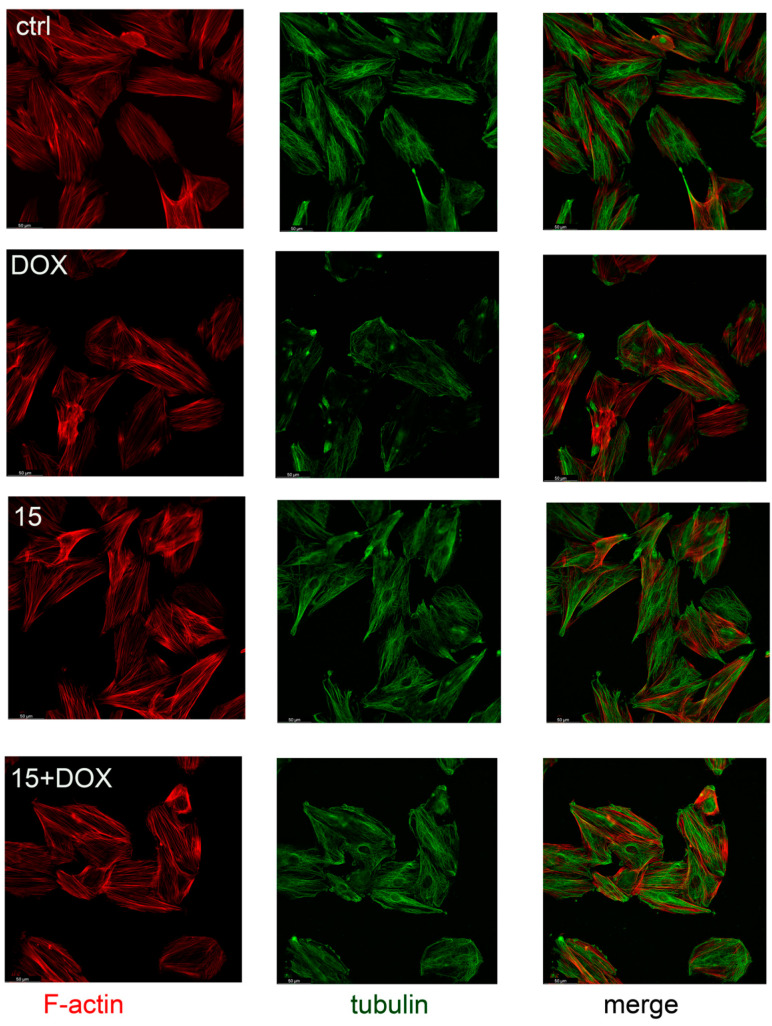
Compound **15** protected rat cardiomyocytes’ (H9c2) cytoskeletons from DOX-induced damage. Pictures show the changes in the actin and microtubule cytoskeletons of rat cardiomyocytes (H9c2) preincubated with compound **15** at a concentration of 25 μM for 8 h and then exposed to DOX at a concentration of 0.5 μM for next 16 h. Controls were: cells exposed only to the culture medium (ctrl), cells exposed only to DOX (DOX), and cells exposed only to compound **15**. Bar = 50 μm.

The bidirectional relationship between heart function and cardiomyocytes’ morphology exists, as tissue remodeling and myocardial contractility dysfunction have been shown to be the result of changes in the cytoskeleton [30]. In addition, the cytoskeleton is also involved in cell migration and myocardial regeneration processes [31]. In the present study, rat cardiomyocytes were preincubated in the presence of compound **15**, and then DOX (0.5 µM) was added for the next 16 h. After incubation, cells were fixed and immunostained with tubulin antibody labeled with Alexa 488, and counterstained with phalloidin labeled with TRITC for cytoskeleton visualization. Our study shows that compound **15** protected cardiomyocytes’ cytoskeletons against DOX damage. DOX used alone induced visible cytoskeleton rearrangement (Figure 4).

### 2.5. Safety Assessment of CA Derivatives

**Figure 5:**
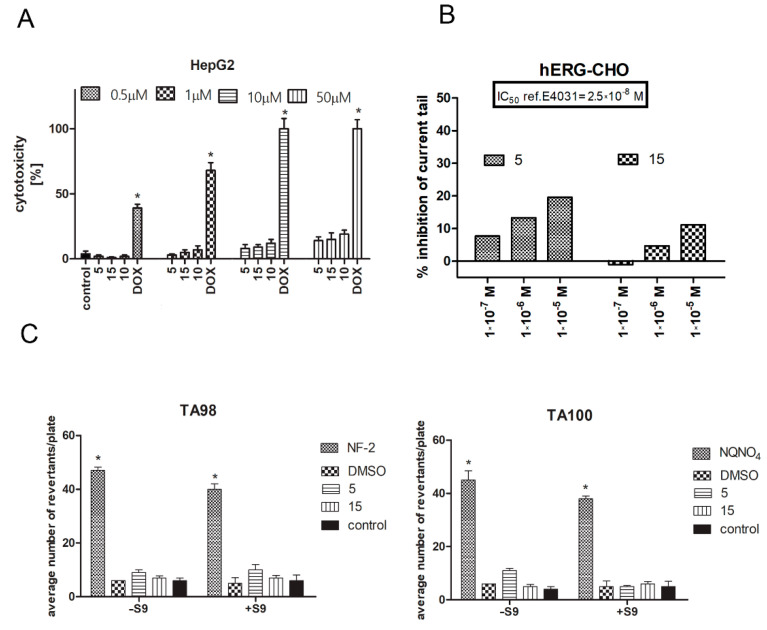
Safety assessment of CA derivatives. Hepatotoxicity analysis was conducted on the HepG2 cell line (**A**). Cells were incubated in the presence of analyzed compounds for 24 h, then the CytoTox-Glo assay was performed. Graph represents the % of cytotoxicity ± SEM of six replicates of three independent experiments. DOX was used as a reference standard. Statistical significance (*) was calculated relative to control (*p* < 0.05). ADME-Tox cardiotoxicity was investigated using the hERG-CHO cell line in an automated patch clamp assay performed by Eurofines. The degree of inhibition (%) was obtained by measuring the tail current amplitude, which was induced by a 1-s test pulse to –40 mV after a 2-spulse to +20 mV, before and after incubation with the compounds (**5**, **15**) (the difference in current was normalized to control and multiplied by 100 in orderto obtain the percent of inhibition). Compound E4031 was used as a reference standard. The experiment was performed in triplicate (**B**). Mutagenicity of compounds **5** and **15** in the absence (–S9) or presence (+S9) of the S9 fraction using the Ames test. Graphs represent the average number of revertants/plate of TA98 and TA100 bacteria strains growing after incubation with the compounds ± SEM of nine replicates of three independent experiments. Statistical significance (*) was calculated relative to control using the Mann–Whitney test (*p* < 0.05). Positive controls used for the MPF protocol were 2-nitrofluorene (2-NF) (TA98, –S9) and 4-nitroquinoline-N-oxide (4-NQO) (TA100, –S9); DMSO was used as the negative control (**C**).

The CytoTox-Glo cell membrane integrity test was used to exclude hepatotoxic activity of CA derivatives. HepG2, a hepatocellular carcinoma cell line, was incubated in the presence of compounds **5**, **10**, and **15** for 24 h at a concentration range of 1–50 µM, and then cytotoxicity analysis was performed. DOX was used as a reference. The results clearly demonstrate that the analyzed CA derivatives are completely safe and do not induce cell membrane damage in the human HepG2 cell line. In contrast, DOX induced a strong cytotoxic effect even at low concentration (0.5 µM) (Figure 5A).

CA derivatives **5** and **15** were investigated in the hERG (hERG-CHO, automated patch clamp) assay. Since 2005, an hERG channel is a part of the preclinical safety panel of new drugs during pharmaceutical research and development [32]. The experiment was performed in accordance with Eurofins’ validation standard operating procedure. E4031 was used as a reference standard in the study. The IC_50_ value for the reference was 2.5 × 10^−8^ M. Analysis shows that CA derivatives investigated in three concentrations (10^−5^, 10^−6^, and 10^−7^ M) do not significantly inhibit the tail current, thus excluding its cardiotoxic activity in relation to the hERG channel (Figure 5B).

In the present study, an Ames MPF 98/100-1 + S9 + KP microplate format mutagenicity assay (Xenometrix, Allschwil, Switzerland) was used to evaluate the mutagenicity of two selected CA derivatives (**5** and **15**). Growth, exposure, and indicator media, liver S9 fraction, S9-NADP, S9-G-6-P, and positive control chemicals, as well as *Salmonella typhimurium* strains, were included in the kit from Xenometrix. According to the obtained results, there were no doses of test compounds **5** and **15** with more than a twofold induction over the baseline, and a dose-dependent response was not observed in either the absence or presence of metabolic activation (Figure 5C). Therefore, compounds **5** and **15** were nonmutagenic in the absence and presence of metabolic activation.

## 3. Discussion

DOX is an anthracycline antibiotic widely used alone or in combination to treat hematological and solid tumors [33]. Despite its widespread use, DOX therapy is often limited by its major side effects, one of which is cardiotoxicity [34]. Cardiotoxicity significantly affects patients’ outcomes and seriously limits their oncological therapeutic opportunities. Childhood cancer survivors treated with DOX have a high risk of heart failure as teenagers [35]. The pathogenesis of cardiotoxicity and anthracycline-induced cardiomyopathy have not been fully elucidated, and are extensively reviewed [5,36]. The main mechanism is attributed to the reactive oxygen species (ROS) and complex ROS-dependent reactions, such as lipid peroxidation, DNA damage, mitochondrial dysfunction, apoptosis, and alterations in iron metabolism through iron–DOX complex formation or interference in the activity of the proteins that transport and bind intracellular iron. It has been suggested that downstream molecular signal transduction pathways in response to reactive oxygen species play pivotal roles maintaining cardiomyocyte homeostasis. The impact on topoisomerase-IIβ as a cause of DOX cardiotoxicity has also been described [37]. More recently, the DOX metabolism, dysregulation of autophagy in the myocardium, and cardiomyocyte senescence were shown to play a contributing role in DOX-induced cardiomyopathy [38]. To date, many combination therapies with cardioprotective agents have been proposed to improve DOX treatment efficacy and safety. ACE inhibitors, beta blockers, reduced glutathione, oleanolic and ursolic acids, and antioxidants have been thoroughly studied [27,39,40]. Many of the mentioned drugs have adverse effects and, therefore, the choice of the cardioprotective agent during cancer treatment should be carefully evaluated [41]. The search for new molecules with multidirectional cardioprotective activity is of great importance to medicine.

Cinnamic acid derivatives are known for their anti-inflammatory, antibacterial, and cytoprotective properties [42]. Cinnamic acid has also shown a protective effect against myelosuppression inhibition and oxidative stress induced by cyclophosphamide in mice. It also prevented against cisplatin-induced splenotoxicity in rat models [43,44]. Moreover, cardioprotective activity of p-coumaric acid, which belongs to the hydroxycinnamic acid family, has also been confirmed [21,45]. We designed and synthetized CA derivatives that possess chemosensitizing activity in DOX-treated lung cancer cells. Their mechanism of action is connected to CBR1 enzyme inhibition and inhibition of DOXol formation. Here we present the most promising CA derivatives tested in our group (**5**, **10,** and **15**) for cardioprotective activity [22]. Limiting the production of DOXol may protect the heart muscle from the adverse effects of DOX. In our study, two cellular models—H9c2 rat cardiomyocyte cells, and human hiPSC-CMs—were used. These cells show biochemical properties of cardiac tissue, and are similar to primary cardiomyocytes. However, experiments carried out on the H9c2 cell line do not predict human-specific sensitivity. The hiPSC (hiPSC-CMs) model of differentiated cardiomyocytes offers a novel approach to cardiac safety assessment in drug discovery [46]. The technology of hiPSCs has revolutionized drug research, enabling the creation of in vitro models using cells isolated directly from patients [47]. hiPSC-CMs were recognized as an appropriate platform for identifying and characterizing the basis of DOX-induced cardiotoxicity [48]. DOX, apart from influencing the morphology and functionality of hiPSC-CMs, has a strong influence on protein expression, and induces changes at the level of the proteome, transcriptome, and the regulatory microRNA network [46]. However, there is scant data describing the use of hiPSC-CMs in the context of searching for compounds with cardioprotective effects [49].

Cardioprotective effects can be mediated by the activation of various signaling pathways in cardiomyocytes. Enzymatic chitosan hydrolysates have been shown to prevent DOX-induced oxidative stress and apoptosis by activating the Nrf2/ARE pathway [50]. Luteolin 7-O-glucoside protects H9c2 cells by activating the PTEN/Akt and ERK pathways [51]. Cardamonin activates the Nrf2-related cytoprotective system, and protects the heart from oxidative damage, apoptosis, and inflammatory injury [52]. In order to check the initial cardioprotective activity of compounds **5**, **10,** and **15**, a comprehensive assessment of the cellular viability, the level of reactive oxygen species, and the condition of the mitochondria was conducted. In the present study, cardiomyocytes’ viability was significantly reduced in the DOX group compared with the control group, which indicated that DOX displayed an inhibitory effect on cardiomyocyte viability. Furthermore, compared with the DOX group, hiPSC-CMs treated with DOX–CA derivatives displayed significantly increased cell viability, suggesting that CA derivatives attenuated the inhibitory effects of DOX on myocardial cells’ survival. The observed effect on H9c2 cardiomyocytes was smaller.

Reports show that CA derivatives can reduce the generation of oxygen free radicals in order to decrease cell damage [53]. The present study demonstrated that DOX reduced the ability of cells to resist oxidation, whereas CA derivatives reduced oxidative stress in cardiomyocytes compared with the DOX group. The free radical scavenging activity of cinnamic acid derivatives was previously reported [20,21]. This might protect the cardiomyocytes from a redox imbalance produced by DOX [54]. Next, we looked at the mitochondrial condition. Mitochondrial dysfunction results in impaired mitochondrial electron transport chain activity, interruption of ion homeostasis, and ROS production. DOX induces changes in mitochondrial morphology as well as mitochondrial membrane potential. CA derivatives protect mitochondria against DOX-induced damage. The effect of compound **10** is debatable as it was the only compound that did not show a significant protective effect.

The induction of cardiomyocyte apoptosis is another pathogenic mechanism in DOX-induced acute cardiotoxicity. Intrinsic apoptosis is the main cause of myocardial tissue loss of function [55]. DOX cardiotoxicity reflected by apoptosis in cardiomyocytes eventually leads to irreversible heart injury. Vanillin ameliorates DOX-induced toxicity in H9c2 cells by reducing DOX-related apoptosis via the attenuation of nuclear shrinkage and deformity and increase of cleaved caspase-3 and PARP1 [56]. All analyzed CA derivatives protected cardiomyocytes against caspase-dependent apoptosis in both cellular models. However, detailed analyses are needed in order to evaluate the exact molecular mechanism of apoptosis.

The effect of CA derivatives on DOX-induced inhibition of the migratory activity of rat cardiomyocytes was evaluated using a wound-healing model. The analysis of migration activity in that model was previously described in order to assess the protective properties of hyaluronic acid against H₂O₂-induced damage to H9c2 cells, and to investigate the role of microRNA-19b in the process of myocardial remodeling in neonatal rat cardiac fibroblasts (NRCFs) [57]. Compounds **5** and **15** abolished the DOX effect and stimulated wound overgrowth compared to control conditions. Neither **10** nor **QUE** protected cardiomyocytes from DOX-induced migratory inhibition. Since the cytoskeleton is responsible for cell migration, it was decided to check whether the tested compounds prevent cytoskeleton damage caused by DOX. DOX used alone induces cytoskeleton remodeling, with the formation of a ring in the cortical layer of the cytoplasm at the periphery of the cell, and disruption of stress fibers, resulting in deterioration of cell adhesion and increased cell detachment [58]. DOX also affects the stability of the cytoskeleton by inhibiting actin polymerization. The ability of **QUE** to prevent DOX-induced cytoskeleton damage was described. Chen (2013) indicated that pretreatment with **QUE** modulated the expression of cytoskeleton proteins and protected the actin cytoskeleton in H9c2 cells against DOX damage [59]. In our study, we observed that DOX used alone reduced cell adhesion, and induced actin accumulation in the nucleus and fiber fragmentation. Protective properties against DOX-induced damage were observed for compound **5**, **15,** and **QUE** (data not shown). Compound **15** showed the most profound effect. The lack of protective activity of compound **10**, in the context of migration, may relate to the cytoskeleton remodeling. However, **QUE’s** lack of protective activity should be carefully evaluated.

The tested derivatives were analyzed for safety in terms of hepatotoxicity, mutagenicity, and interaction with hERG channels. Cinnamic acid and its derivatives have a satisfactory safety profile. However, in the case of chemical modifications, new, active structures should be tested. In the present study we excluded the hepatotoxic and mutagenic activity of CA derivatives. These data are consistent with the literature. Lack of mutagenic activity from cinnamic acid derivatives was shown using the Ames test by Taner (2017) [60]. Similarly, Maistro (2011) showed that cinnamic acid is not genotoxic using the comet test [61]. However, it was confirmed that it has a clastogenic effect related to changes in the structure of chromosomes, which was proven by the micronucleus test [61]. The tested compounds also showed no inhibitory effect on hERG channels. These results exclude frequent side effects of compounds with primary cytoprotective activity proved in vitro models.

## 4. Limitations

The main limitation of the conducted study was the lack of precise data on the influence of the tested compounds on cellular electrophysiological features. The safety studies of the analyzed compounds that involved drug-induced hERG-related cardiotoxicity were investigated using the patch clamp technique (hERG-CHO). From the point of view of future investigation, it would be interesting to perform such analysis in the hiPSC-CM model. The tested compounds induced a slight dose-dependent inhibition of the hERG channel, which raises some concerns. Further analyses, including computational modelling, are needed to finally confirm the safety of CA derivatives. In addition, we examined the safety of the compounds used alone; however, it would be worth checking how they affect the electrophysiological properties of cells in combination with DOX. Moreover, the selection of methods for the safety panel needs to be extended to include methods of testing the frequency of cellular arrhythmia determinants (e.g., EADs and DADs).

## 5. Materials and Methods

### 5.1. Chemistry

Compounds **5** ((R,S)-(E)-N-(1-hydroxy-3-methylbutan-2-yl)cinnamamide), **10** ((E)-3-(4-chlorophenyl)-N-(1-hydroxy-2-methylpropan-2-yl)acrylamide), and **15** ((E)-1-(4-hydroxypiperidin-1-yl)-3-phenylprop-2-en-1-one) were synthesized from (E)-cinnamoyl chloride (**5** and **15**) or (E)-4-chlorocinnamic acid chloride (**10**), and appropriate aminoalkanol: R,S-2-amino-3-methylbutan-1-ol for **5**, 2-amino-2-methylpropan-1-ol for **10**, and 4-hydroxypiperidine for **15**. The N-acylation reactions were carried out in toluene/potassium carbonate solution two-phase system according to previously published procedures [23,24]. Structures and purity of the compounds were confirmed using spectroscopic methods (NMR, LC/MS) as well as RP-HPLC, and additionally with crystallographic studies for compounds **5** and **15**. The detailed synthesis procedures and physiochemical data of the compounds were previously published [23,24,25,26]. The chemical structures of the tested compounds are presented in Table 1.

### 5.2. Cell Culture

Human hepatocellular carcinoma (HepG2, ATCC HB-8065) cells were cultured in Eagle’s Minimum Essential Medium (EMEM, ATCC, Manassas, VA, USA). Rat cardiomyocytes (H9c2, ATCC CRL-1446) were cultured in Dulbecco’s Modified Eagle’s Medium (DMEM, ATCC). Both were prepared with the addition of a 1% antibiotic mixture (penicillin and streptomycin) and 10% fetal bovine serum (FBS; Gibco, Life Technologies, Waltham, MA, USA). CA derivatives were applied from DMSO stock solutions and diluted in the culture medium to the working concentrations. The influence of the DMSO on the viability of cells in the highest applied CA concentration was checked, and it turned out to be non-toxic. Doxorubicin (Cayman, Ann Arbor, MI, USA, purity 99% HPLC) was applied to the culture medium in the working concentrations from freshly made stock solution in DMSO (500 µM).

### 5.3. Differentiation of Human iPSC-Derived Cardiomyocytes

hiPSCs were cultured in Geltrex^TM^-coated 12-well plates (Geltrex^TM^ LDEV-Free hESC-Qualified Reduced Growth Factor Basement Membrane Matrix, Thermo Fisher Scientific, Waltham, MA, USA) in Essential 8^TM^ medium (Thermo Fisher Scientific; E8 medium) and passaged using 0.5 mM EDTA. We added 10 μM ROCK inhibitor Y-27632 (Abcam, Cambridge, United Kingdom) to the culture medium for the first 24 h after passage. Differentiation towards cardiomyocytes was performed according to the published protocol [62,63]. Briefly, hiPSCs were seeded in Geltrex^TM^-coated 24-well plates and cultured in E8 until reaching 90% confluency. Cells were then stimulated with CHIR99021 (Sigma-Aldrich, Saint Louis, MO, USA) in RPMI1640 medium (Biowest, Riverside, MO, USA) containing 2% B-27 supplement without insulin (Thermo Fisher Scientific; RMPI/B27-ins). Twenty-four hours later (day one), the medium was replaced with fresh RPMI/B27-ins. On day 3, cells were stimulated for 48 h with IWR-1 (Sigma-Aldrich), after which fresh RMPI/B27-ins was added. On day 7, the medium was replaced with RPMI1640 containing 2% B-27 supplement (Thermo Fisher Scientific; RPMI/B27), and cells were further cultured for an additional 2 weeks with RPMI/B27 changed every 3rd day. The cells used in the study were of maturation age: 25–35 days post-differentiation [64,65]. All cells were maintained in a humidified tissue culture incubator at 37 °C and 5% CO_2_.

### 5.4. Preparation of Human iPSC-Derived Cardiomyocytes for Experiments

To prepare hiPSC-CMs for further analyses, cells were washed with PBS twice, then PBS solution was aspirated, and Multi Tissue Dissociation Kit 3 (400 µL) was used to detach the cells from the dish. After 10 min incubation at 37 °C, culture medium with 20% FBS was added. Cells were gently detached by pipetting. The density of the cells was determined using a Burker chamber. The appropriate number of cells was seeded in 96-well plates coated with Geltrex^TM^ for the designed experiments.

### 5.5. Cytotoxicity Analysis

Cell viability was measured using 3-(4,5-dimethylthiazol-2-yl)-2,5-diphenyltetrazolium bromide (MTT) assay. Cells were seeded at a density of 1 × 10^4^ in 96-well plates. After 24 h, cells were preincubated with the tested compounds—**5**, **10**, **15** (25 µM), and quercetin (**QUE**, 20 µM) as a reference—for 5 h. Then, the medium was removed, and cells were incubated with DOX (5 or 10 µM) for the next 90 min. Following cell exposure, 10 µL of MTT reagent (Sigma Aldrich) was added to each well. After 4 h of incubation (37 °C, 5% CO_2_), the medium was aspirated, and formazan produced in cells appeared as dark crystals in the bottom of the wells. Next, 100 µL of formazan dissolvent (DMSO) was added to each well. Then, the optical density (OD) of the obtained solution at 570 nm was determined using a plate reader (Spectra Max iD3, Molecular Devices, San Jose, CA, USA). Each individual experiment was repeated at least three times.

### 5.6. ROS-Glo™ H_2_O_2_ Assay

The ROS-Glo™ H_2_O_2_ assay from Promega (Mannheim, Germany) was performed in accordance with the manufacturer’s instructions. The incubation procedure was analogous to that used for cytotoxicity assay. Cells were grown for 24 h. Afterwards, the medium was replaced with 100 µL EMEM medium containing 2% FBS and 1% Pen/Strep. Subsequently, 5 µL of test compound stock solution (DMEM or menadione as positive control) and 20 µL H_2_O_2_ substrate solution were added and incubated for 5 h. In addition, the amount of ROS was also measured, both in medium alone and in medium plus test compound (without cells). Finally, 100 µL of ROS-Glo™ Detection Solution was added to each well, incubated for 20 min, and luminescence was recorded using a plate reader (Spectra Max ID3, Molecular Devices).

### 5.7. Mitochondrial Morphology

H9c2 cells were seeded at a density of 1 × 10^4^ cells/well in 24-well plates with coverslips located at the bottom of the wells. Cells were grown for 24 h. After incubation in the presence of DOX, QUE, CA, QUE–DOX, or CA–DOX, cells were stained with MitoTracker Deep Red FM (Invitrogen, Carlsbad, CA, USA) according to the manufacturer’s protocol. After determining the lowest concentration (0.1 µM) of dye necessary to acquire high signal-to-noise ratio images, cells were stained for 15 min at 37 °C with the Mito Tracker Deep Red in phenol-red-free DMEM supplemented with 1% FBS (the concentration of Mito Tracker was 1 µM). The media were then replaced for imaging.

### 5.8. Mitochondrial Membrane Potential Assay (JC1)

The 5,5′,6,6′-tetrachloro-1,1′,3,3′-tetraethylbenzimidazolylcarbocyanine iodide assay kit JC1 (Abcam) was used to assess mitochondrial membrane potential in H9c2 cardiomyocytes incubated in the presence of the analyzed compounds and the reference control (FCCP), according to the manufacturer’s instructions. The plates were then incubated at 37 °C for 10 min in the dark after the addition of 100 μL of 1 × JC-1 reagent to the wells. Next, plates were washed twice with dilution buffer solution. Fluorescence was measured using a plate reader (Spectra Max iD3, Molecular Devices) set to an exc/em wavelength of 535/590 nm (aggregate) or 475/530nm (monomer). Aggregate/monomer ratios were plotted for four technical replicates for each condition. Aggregate/monomer ratios decreased, as indicated by the depolarized MMP.

### 5.9. Caspase-Glo 3/7 Assay

The ability of CA derivatives to prevent apoptosis was evaluated by measuring the caspase-3/7 activity on H9c2 and hiPSC-CMs using the Caspase-Glo 3/7 assay kit (Promega). Cells were seeded at a density of 1 × 10^4^ cells/well in 96-well plates and were allowed to adhere overnight. These cells were preincubated with the compounds (**5**, **10**, **15**, and **QUE**) for 24 h and then DOX was added to each well at a concentration of 0.5 µM for the next 24 h. After treatment, the Caspase-Glo^®^ 3/7 assay was prepared according to the manufacturer’s guidelines, and 100 μL of the reagent was added per well and incubated for 1 h at room temperature in the dark. Following this incubation, the luminescence was measured using a microplate reader (Spectra Max ID3, Molecular Devices). Additionally, an experiment with the caspase inhibitor Z-VAD-FMK (20 µM), which irreversibly binds to the catalytic site of caspase proteases and inhibits caspase-dependent induction of apoptosis, was performed. The caspase inhibitor was co-incubated with cells during standard experiments. The data were analyzed and expressed as the percentage of the untreated cells (control).

### 5.10. Wound-Healing Assay In Vitro

Rat cardiomyocytes were seeded in quantities of 1 × 10^5^ per well in 12-well plates (Falcon, Corning Life Sciences, Tewksbury, MA, USA) and cultivated overnight in order to achieve 100% confluence in the presence of CA derivatives and quercetin (preincubation). Artificial wounds were made manually with a pipette tip, after which the culture medium was replaced with a fresh one containing the desired concentration of DOX. Images were captured with a Leica DMI 6000B fully motorized inverted microscope equipped with modulating contrast, an environmental chamber, and a DFC360 FX CCD camera (Leica, Wetzlar, Germany). Time-lapse imaging was performed under the control of LAS X software (Leica Microsystems, Wetzlar, Germany) every 10 min for 16 h in multiple fields of view. Image analysis was conducted in Fiji distribution of ImageJ software [66]. The wound area was outlined automatically using MRI Wound Healing Tool, measured and calculated as a difference between a given time step and the initial one. Results were averaged with 30 min time intervals for at least 10 fields of view from 2 separate experiments.

### 5.11. Immunostaining

To visualize the actin cytoskeleton, cells were seeded on glass coverslips inserted into 12-well plates at density of 1×10^4^ cells/cm^2^ and cultivated for 24 h. The culture medium was then replaced with fresh media without compounds (controls) or with compound **15** alone. After the next 24 h, the preincubation medium was removed and replaced with fresh medium containing DOX (0.5 µM) for 16 h. Then, the cells were washed with PBS with Ca^2+^ and Mg^2+^ ions, fixed in 3.7% paraformaldehyde/PBS at room temperature and permeabilized with 0.1% Triton X-100/PBS for 6 min. The cells were then incubated with 1% BSA/PBS for 45 min, followed by the incubation with monoclonal mouse antibody against tubulin (Sigma-Aldrich) at RT. Afterwards, cells were washed with PBS, incubated with the corresponding Alexa Fluor 488 conjugated goat anti-mouse IgG antibody (clone A11001, Sigma-Aldrich), and counterstained with phalloidin (500 ng/mL, Sigma-Aldrich).

### 5.12. Hepatotoxicity

The CytoTox-Glo cell membrane integrity test (Promega) was used for cytotoxicity testing. The assay was performed according to the manufacturer’s protocol. HepG2 cells were seeded at a density of 1 × 10^4^ cells/well in white 96-well culture plates and incubated for 24 h. Then, the cells were incubated in the presence of the analyzed compounds (**5**, **10**, and **15**) for 24 h. Triton X-100 was used as a positive control, and the solvent of compounds (0.01% DMSO) was applied as a negative control. Doxorubicin, a cytotoxic agent, was used as a reference. CytoTox-Glo™ reagent was added to each well. Luminescence signals were measured with a microplate reader (Spectra Max ID3, Molecular Devices) (signal 1). Next, lysis solution was added and incubated for 15 min to detect the total signal, and luminescence was measured again (signal 2). The percentage of living cells was calculated by subtracting signal 1 from signal 2. The signal of control condition was set to 100%, and the signal of TritonX-100 to 0% living cells. Cytotoxicity was calculated as 100-A, where A is the viability of the cells in the analyzed sample (relative to controls).

### 5.13. hERG (hERG-CHO, Automated Patch Clamp)

hERG (hERG-CHO, automated patch clamp) was used to assess the cardiotoxicity of compounds **5** and **15**. Analyses were performed on the hERG CHO-K1 cell line. In each experiment, if applicable, the respective reference compound was tested concurrently with **5** and **15**, and the data were compared with historical values determined at Eurofins. The experiment was performed in accordance with Eurofins validation standard operating procedure. The degree of inhibition (%) was obtained by measuring the tail current amplitude, which is induced by a 1-s test pulse to −40 mV after a 2-s pulse to +20 mV, before and after drug incubation (the difference current was normalized to controls and multiplied by 100 in order to obtain the percentage inhibition). Concentration (log) response curves were fitted to a logistic equation (three parameters assuming complete block of the current at very high test compound concentrations) to generate estimates of the 50% inhibitory concentration (IC_50_). The concentration response relationship of each compound was constructed from the percentage reductions of current amplitude by sequential concentrations.

### 5.14. Mutagenicity—Ames Test

During the procedure, bacteria (*Salmonella typhimurium*) were exposed to 3 different concentrations of a test compound for 90 min in a medium containing sufficient histidine to support cell growth. Then, the cultures were transferred to pH indicator medium lacking histidine and aliquoted into 384-well plates. Within 48 h, cells that had undergone reversion to amino acid prototrophy grew into colonies. Bacterial metabolism reduces the pH of the medium, changing the color of the well from purple to yellow. The following strains were used in the study: *Salmonella typhimurium* TA98 and TA100 both indicate frame shifts and base-pair mutations. The test procedure was provided by Xenometrix (Allschwil, Switzerland). TA98 and TA100 bacterial strains were grown overnight in exposure medium and in the presence of compounds **5** and **15** at final concentrations of 0.1, 0.2, and 0.5 mM in 24-well plates for 90 min at 37 °C in the absence (–S9) or presence (+S9) of 4.5% phenobarbital/β-naphthoflavone-induced rat liver S9. Compounds **5** and **15** were dissolved in DMSO. After the preincubation, the cultures were diluted in the indicator medium and the contents of each 24-well culture were distributed into 384-well plates and incubated further for 48 h at 37 °C. After the exposure, the number of wells containing bacteria was scored by counting yellow wells. Positive controls used for the MPF protocol were 2-nitrofluorene (2-NF) at 2 µg/mL (TA98, –S9) and 4-nitroquinoline-N-oxide (4-NQO) at 0.1 µg/mL (TA100, –S9); DMSO was used as the negative control. To evaluate the test results, the following criteria were used: The fold increase of revertants relative to the solvent control was determined by dividing the mean number of positive wells at each dose by the solvent control at baseline. The solvent control at baseline was defined as the mean number of positive wells in the solvent control plus one standard deviation (SD). When an increase of more than twofold relative to the baseline at more than one dose with a dose–response was observed, the sample was classified as positive, whereas when no response over two times the baseline and no dose–response was stated, the sample was negative.

### 5.15. Statistical Analysis

Data are expressed as mean ± SEM. Statistically significant values were compared using the Brown–Forsythe and Welch’s ANOVA tests, along with the post-hoc unpaired t-test with Welch’s correction, and the Mann–Whitney test, using GraphPad Prism 5 software (GraphPad Software, San Diego, CA, USA); *p*-values of less than 0.01/0.05 were considered statistically significant.

## 6. Conclusions

In conclusion, we presented new beneficial effects of three CA derivatives with previously proven DOX-chemosensitizing activity [22]. Apart from the beneficial antitumor activity, these derivatives could protect myocardial cells against the toxic effects of DOX. All of the tested compounds (**5**, **10**, and **15**) showed protective effects. However, compound **15** ((E)-1-(4-hydroxypiperidin-1-yl)-3-phenylprop-2-en-1-one) was the clear leader. Its cardioprotective effect was manifested in all of the examined molecular aspects. Its safety was confirmed using gold standard tests. Compound **15** can serve as a starting point for the synthesis of structural analogs with a beneficial protective effect. The double activity observed in the CA derivatives group highlights the potential use of these compounds in the future as promising drug candidates. Further molecular studies need to be conducted, which will shed light on the mechanism by which CA derivatives reduce the cardiotoxic effects of DOX.

## Figures and Tables

**Table 1:** Chemical structures of the tested compounds.

Compound 5	Compound 10	Compound 15
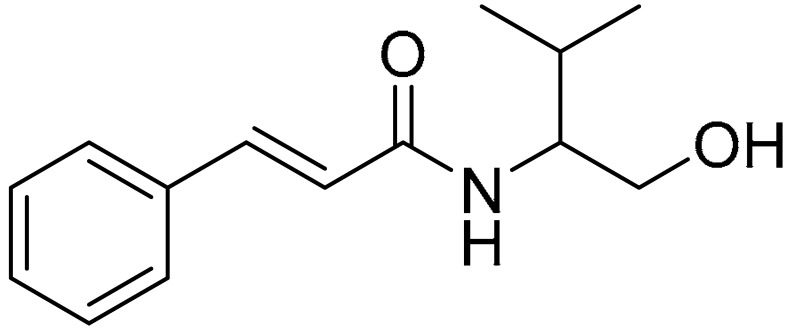	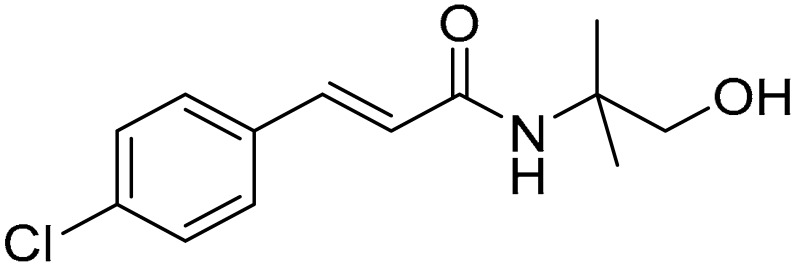	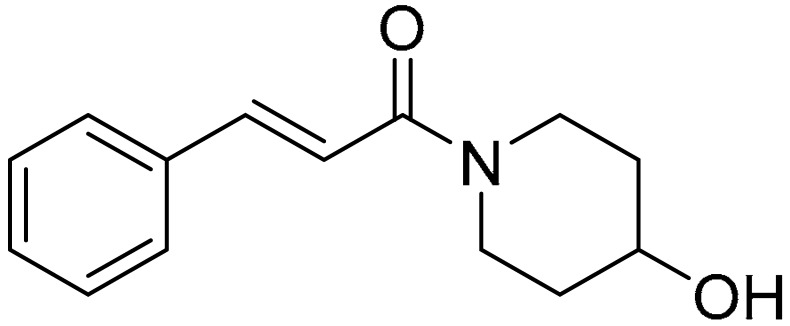

## Data Availability

The data presented in this study are available within the article text and figures. Data are available from the corresponding author upon request.
